# Comparison of clinical laboratory tests between bacterial sepsis and SARS-CoV-2-associated viral sepsis

**DOI:** 10.1186/s40779-020-00267-3

**Published:** 2020-08-04

**Authors:** Chao Ren, Ren-Qi Yao, Di Ren, Ying Li, Yong-Wen Feng, Yong-Ming Yao

**Affiliations:** 1 Department of Critical Care Medicine, the Second People’s Hospital of Shenzhen, Shenzhen, 518035 Guangdong China; 2 Department of Critical Care Medicine, the Third People’s Hospital of Shenzhen, Shenzhen, 518020 Guangdong China; 3 Trauma Research Center, the Fourth Medical Center and Medical Innovation Research Department of the Chinese PLA General Hospital, Beijing, 100048 China

**Keywords:** SARS-CoV-2, COVID-19, Sepsis, Bacteria, Virus, Infection, Host response

## Abstract

Sepsis is a life-threatening condition that is characterized by multiple organ dysfunction due to abnormal host response to various pathogens, like bacteria, fungi and virus. The differences between viral and bacterial sepsis are indeed of great significance to deepen the understanding of the pathogenesis of sepsis, especially under pandemics of SARS-CoV-2 infection.

Dear editor,

The pandemics of severe acute respiratory syndrome coronavirus 2 (SARS-CoV-2) infection have become a world health crisis that cause significant loss of life. The dysregulated host response to SARS-CoV-2 appears to be associated with the severity and poor outcomes of coronavirus disease 2019 (COVID-19) patients [1], hinting the critical involvement of SARS-CoV-2-induced sepsis based on Sepsis 3.0 definition [2–4]. The development of sepsis accounts for one of the leading causes of death in patients admitted to intensive care units (ICU), but evidences on viral sepsis remain scarce in current clinical practices, let alone its differences with bacterial sepsis. Given the pandemics of SARS-CoV-2 infection [5], the differences between viral and bacterial sepsis are indeed of great significance to deepen the understanding of the pathogenesis of sepsis.

In this study, we obtained the clinical data from two cohorts up to May 13, 2020, including 41 critically ill COVID-19 patients from the Third People’s Hospital of Shenzhen and 194 non-COVID-19 patients admitted to ICU of the Second People’s Hospital of Shenzhen, China. Demographic characteristics, comorbidities, and laboratory findings on ICU admission and clinical outcomes were collected. Sequential Organ Failure Assessment (SOFA) and Acute Physiology and Chronic Health Evaluation II (APACHE II) scores were calculated within the first 24 h since ICU admission. The bacterial and viral sepsis were identified by blood culture and metagenomic next-generation sequencing.

Twenty-one patients with SARS-CoV-2-induced sepsis and 46 patients with bacterial sepsis were finally recruited (Additional file 2). The median age was 64.0 years (IQR, 60.5–68.0) and 65.5 years (IQR, 49.3–77.3) for patients of SARS-CoV-2-induced sepsis and bacterial sepsis, respectively (Additional file 1). The prognostic scoring system, including SOFA [6.0 (IQR, 4.0–9.0) vs. 4.0 (IQR, 3.5–5.0), *P* = 0.01] and APACHE II [17.0 (IQR, 13.0–20.3) vs. 8.0 (IQR, 6.5–9.5), *P* < 0.001] were consistently higher among patients with bacterial sepsis than those with SARS-CoV-2-induced sepsis. Meanwhile, ICU mortality rates were significantly higher in patients with bacterial sepsis than those with viral sepsis [34.8% (16/46) vs. 4.8% (1/21), *P* = 0.013].

As presented in Table 1, absolute counts of T lymphocytes, cytotoxic T lymphocytes (Tc), and helper T lymphocytes (Th) were significantly lower among patients with SARS-CoV-2-induced sepsis at ICU admission, while elevated inflammation-related parameters, C-reactive protein (CRP) as an example, were observed in this cohort compared to patients with bacterial sepsis. In addition, obvious differences in organ functional parameters were noted between the two cohorts, including significantly increased levels of creatine kinase-MB and NT-pro BNP, and decreased albumin level in patients with developed bacterial sepsis.

**Table 1:** Comparison of laboratory findings between SARS-CoV-2- and bacteria-induced septic patients admitted to ICU

Item	Normal range	Total (*n* = 67)	SARS-CoV-2-induced sepsis (*n* = 21)	Bacteria-induced sepsis (*n* = 46)	*P*
**Blood routine test**					
White blood cell counts (× 10^9^/L)	3.5–9.5	9.6 (6.1–15.9)	7.0 (4.7–10.9)	11.7 (6.6–17.3)	0.007
Neutrophil counts (× 10^9^/L)	1.8–6.3	9.0 (4.3–13.9)	5.8 (3.6–8.9)	11.1 (5.5–15.3)	0.003
Lymphocyte counts (× 10^9^/L)	1.1–3.2	0.7 (0.5–1.0)	0.6 (0.6–0.8)	0.7 (0.5–1.0)	0.68
Neutrophil to lymphocyte ratio	NA	11.0 (4.8–21.5)	9.5 (4.5–13.1)	12.2 (6.0–24.6)	0.094
Monocyte counts (× 10^9^/L)	0.1–0.6	0.4 (0.2–0.8)	0.4 (0.2–0.5)	0.5 (0.2–0.9)	0.311
Platelet counts (× 10^9^/L)	125.0–350.0	186.0 (141.0–262.0)	181.0 (148.5–237.5)	191.0 (130.0–284.8)	0.71
Haemoglobin [g/L, mean (SD)]	130–175	113.7 (26.0)	131.5 (17.4)	105.6 (25.4)	< 0.001
Hematocrit (%)	40.0–50.0	33.8 (28.6–37.9)	37.3 (34.9–40.4)	30.8 (25.6–36.0)	< 0.001
**Coagulation function**					
Prothrombin time (s)	10.5–13.5	13.0 (11.9–14.1)	13.4 (12.3–13.9)	12.9 (11.8–14.5)	0.665
Activated partial thromboplastin time (s)	21.0–37.0	33.9 (29.1–40.4)	34.7 (32.5–40.2)	32.1 (29.0–41.6)	0.402
International normalized ratio	0.8–1.3	1.1 (1.0–1.2)	1.0 (0.9–1.1)	1.1 (1.0–1.3)	0.002
D-dimer (μg/L)	0–1.5	3.1 (1.6–8.0)	1.1 (0.7–2.8)	4.4 (2.6–9.6)	< 0.001
**Blood biochemistry**					
Albumin, median [g/L, mean (IQR)]	40.0–55.0	30.1 (25.6–33.7)	34.6 (31.3–36.5)	26.8 (24.2–31.6)	< 0.001
Alanine aminotransferase(U/L)	9.0–50.0	38.0 (27.8–60.1)	33.5 (22.0–54.3)	43.0 (28.8–70.0)	0.241
Aspartate aminotransferase (U/L)	15.0–40.0	48.4 (31.8–91.3)	47.0 (32.6–69.5)	48.5 (29.3–100.8)	0.567
Total bilirubin (μmol/L)	0.21.0	15.7 (10.7–22.2)	20.7 (12.8–27.6)	15.4 (8.6–21.0)	0.025
Serum creatinine (μmol/L)	57.0–111.0	77.0 (50.2–124.4)	75.1 (56.3–92.7)	77.0 (48.3–155.1)	0.727
Blood urea nitrogen (mmol/L)	3.6–9.5	7.1 (5.4–9.6)	6.6 (5.5–8.3)	7.4 (5.3–12.8)	0.151
Creatine kinase MB form (U/L)	0–5.0	2.0 (1.1–5.0)	0.8 (0.4–1.9)	2.8 (2.0–7.6)	< 0.001
NT-pro BNP (pg/ml)	0–125.0	1778.5 (223.8–6170.5)	133.5 (71.8–772.0)	3298.5 (473.8–7077.5)	< 0.001
Lactate dehydrogenase (U/L)	120.0–350.0	749.0 (427.0–1290.0)	693.0 (399.0–883.5)	936.5 (490.8–1535.5)	0.089
**Arterial blood gas**					
Sodium (mmol/L)	135.0–145.0	136.0 (132.0–141.0)	135.2 (132.5–141.6)	136.0 (131.8–141.0)	0.797
Potassium (mmol/L)	3.5–5.0	3.6 (3.4–4.1)	3.5 (3.2–3.6)	3.8 (3.5–4.3)	0.02
Chloride (mmol/L)	90.0–110.0	101.0 (97.8–106.0)	101.0 (98.0–106.5)	101.0 (96.8–105.3)	0.695
PaO_2_ (mmHg)	83.0–108.0	81.0 (61.4–99.6)	65.3 (57.5–82.0)	86.5 (64.6–130.0)	0.02
PaCO_2_ (mmHg)	35.0–48.0	33.6 (30.7–40.6)	33.3 (31.4–36.6)	34.0 (28.8–44.9)	0.72
PaO_2_:FIO_2_ (mmHg)	400.0–500.0	162.2 (119.4–219.3)	132.9 (116.2–174.9)	181.3 (119.0–279.9)	0.07
Glucose (mmol/L)	3.9–6.1	9.2 (6.8–13.8)	11.1 (8.3–14.9)	8.8 (5.9–13.0)	0.155
Lactate (mmol/L)	0.5–1.6	2.1 (1.5–2.9)	2.5 (1.6–3.0)	2.0 (1.5–2.6)	0.226
**Immune-related biomarkers**					
Absolute T lymphocyte counts (count/μl)	NA	440.0 (329.5–581.0)	329.5 (313.3–472.3)	492.0 (382.0–720.0)	0.004
Absolute helper T lymphocyte counts (count/μl)	NA	246.0 (188.0–372.0)	201.0 (156.0–264.5)	308.0 (208.0–420.0)	0.034
Absolute cytotoxic T lymphocyte counts (count/μl)	NA	152.0 (102.0–236.0)	101.0 (91.0–153.8)	180.0 (132.0–264.0)	0.003
CD4/CD8 ratio [mean (SD)]	0.9–3.6	1.8 (0.9)	1.9 (0.9)	1.8 (0.9)	0.709
**Inflammation-related biomarkers**					
C-reactive protein (mg/L)	0–10.0	104.7 (30.9–148.4)	119.2 (70.7–154.6)	37.2 (23.8–111.6)	0.008
Procalcitonin (ng/ml)	0–5.0	0.5 (0.2–3.8)	0.2 (0.2–0.3)	1.4 (0.3–5.5)	< 0.001

Data were median (IQR) if not otherwise specified. n (%) referred to the total number of patients with available data. *P* values indicated differences between SARS-CoV-2-induced sepsis and bacteria-induced sepsis, in which *P* < 0.05 was deemed as statistical significance

*SD* Standard deviation, *BNP* Brain natriuretic peptide, *NA* Not applicable

In this study, ICU patients with SARS-CoV-2-induced sepsis and those with bacterial sepsis revealed comparable demographic characteristics, like age, gender distribution, and comorbidities, after rigorous screening processes. However, patients with bacterial sepsis were found with more severe organ dysfunction and poor outcomes when compared with those caused by SARS-CoV-2-induced sepsis, including higher values in SOFA and APACHE II, as well as more ICU deaths. The different patterns of immune responses might be the major cause of the divergent outcomes between viral and bacterial sepsis. We further found that failed homeostasis was characterized in both bacterial and viral sepsis but triggered by different pathogens. In the development of viral sepsis, the loss of T lymphocytes and their subsets was the dominant characteristics of dysregulated immune response, thereby contributing to the imbalance between innate and adaptive immune systems; while excessive inflammatory activation was the main feature of bacterial sepsis, which further resulted in intractable immune suppression and multiple organ dysfunction. This is the first report that compared clinical features and host responses between bacterial and SARS-CoV-2-induced viral sepsis. These findings might not only suggest divergent host responses to bacteria and virus but also provide novel insights into further researches on the development of sepsis with underlying etiology of various pathogens.

## Supplementary information

**Additional file 1.** Appendix Table 1. Baseline characteristics of critically ill patients with SARS-CoV-2- and bacteria-induced sepsis.

**Additional file 2.** Appendix Figure 1. Flow diagram of patient inclusion. COVID-19: Coronavirus disease 2019; SARS-CoV-2: Severe acute respiratory syndrome coronavirus 2; SOFA: Sequential organ failure assessment; ICU: Intensive care unit; LOS: Length of stay; CAP: Community-acquired pneumonia.

## Data Availability

All data were presented in this manuscript or Appendix.

## Abbreviations

COVID-19: Coronavirus disease 2019
CAP: Community-acquired pneumonia
CRP: C-reactive protein
ICU: Intensive care units
SARS-CoV-2: Severe acute respiratory syndrome coronavirus 2
SOFA: Sequential organ failure assessment
APACHE II: Acute physiology and chronic health evaluation II
