# Supplementary material for: Comparison of clinical laboratory tests between bacterial sepsis and SARS-CoV-2-associated viral sepsis
Source: Mil Med Res. 2020 Aug 4;7:36. doi: 10.1186/s40779-020-00267-3 (PMC7399032; doi:10.1186/s40779-020-00267-3)
Supplement: Supplementary file 1 — Additional file 1. Appendix Table 1. Baseline characteristics of critically ill patients with SARS-CoV-2- and bacteria-induced sepsis. [file 40779_2020_267_MOESM1_ESM.docx]

**Appendix Table 1.** Baseline characteristics of critically ill patients with SARS-CoV-2- and bacteria-induced sepsis

| Item | Total  (n=67) | SARS-CoV-2-induced sepsis  (n=21) | Bacteria-induced sepsis  (n=46) | *P* |
| --- | --- | --- | --- | --- |
| **Demographic characteristics** | | | | |
| Age [years, median (IQR)] | 64.0 (56.0-71.0) | 64.0 (60.5-68.0) | 65.5 (49.3-77.3) | 0.556 |
| Female [n (%)] | 21 (31.3) | 6 (28.6) | 15 (32.6) | 0.785 |
| **Comorbidities** [n (%)] | | | | |
| Hypertension | 29 (43.3) | 8 (38.1) | 21 (45.7) | 0.605 |
| Diabetes | 14 (20.9) | 4 (19.0) | 10 (21.7) | >0.99 |
| Chronic bronchitis | 4 (6.0) | 1 (4.8) | 3 (6.5) | >0.99 |
| Malignant tumor | 4 (6.0) | 1 (4.8) | 3 (6.5) | >0.99 |
| Cerebrovascular disease | 4 (6.0) | 1 (4.8) | 3 (6.5) | >0.99 |
| Smoke | 1 (1.5) | 0 (0) | 1 (2.2) | >0.99 |
| **Signs and symptoms** [median (IQR)] | | | | |
| Body temperature (℃) | 37.0 (36.5-38.1) | 37.0 (36.7-38.0) | 37.0 (36.5-38.2) | 0.745 |
| Heart rates (/min) | 100.0 (83.0-125.0) | 80.0 (68.5-88.5) | 113.0 (93.8-130.5) | <0.001 |
| Respiratory rates (/min) | 26 (22-32) | 25 (22.5-26.0) | 27.5 (21.8-34.5) | 0.064 |
| MAP(mmHg) | 83.7 (72.0-99.0) | 85.0 (77.8-105.8) | 80.5 (65.6-99.0) | 0.118 |
| SOFA | 5.0 (4.0-8.0) | 4.0 (3.5-5.0) | 6.0 (4.0-9.0) | 0.01 |
| APACHE II | 13.0 (9.0-18.0) | 8.0 (6.5-9.5) | 17 .0(13.0-20.3) | <0.001 |
| **Prognosis** | | | | |
| Length of stay in hospital[days, median (IQR)], | 31.0 (16.0-42.0) | 38.0 (33.0-44.0) | 22.5 (11.0-38.0) | 0.003 |
| Length of stay in ICU[days, median (IQR)] | 17.0 (8.0-27.0) | 16.0 (8.0-24.5) | 18.5 (8.8-32.5) | 0.317 |
| In-hospital death [n (%)] | 17 (25.4) | 1 (4.8) | 16 (34.8) | 0.013 |

ICU. Intensive care unit; IQR. Interquartile range; SOFA. Sequential organ failure assessment; APACHE II. Acute physiology and chronic health evaluation II. Data were presented as median (IQR) or mean (SD). n (%) referred to the total number of patients with available data. *P* values indicated differences between SARS-CoV-2-induced sepsis and bacteria-induced sepsis patients, in which *P*<0.05 was deemed as statistical significance.
