# Supplementary material for: Comparison of clinical laboratory tests between bacterial sepsis and SARS-CoV-2-associated viral sepsis
Source: Mil Med Res. 2020 Aug 4;7:36. doi: 10.1186/s40779-020-00267-3 (PMC7399032; doi:10.1186/s40779-020-00267-3)
Supplement: Supplementary file 2 — Additional file 2. Appendix Figure 1. Flow diagram of patient inclusion. COVID-19: Coronavirus disease 2019; SARS-CoV-2: Severe acute respiratory syndrome coronavirus 2; SOFA: Sequential organ failure assessment; ICU: Intensive care unit; LOS: Length of stay; CAP: Community-acquired pneumonia. [file 40779_2020_267_MOESM2_ESM.docx]

**
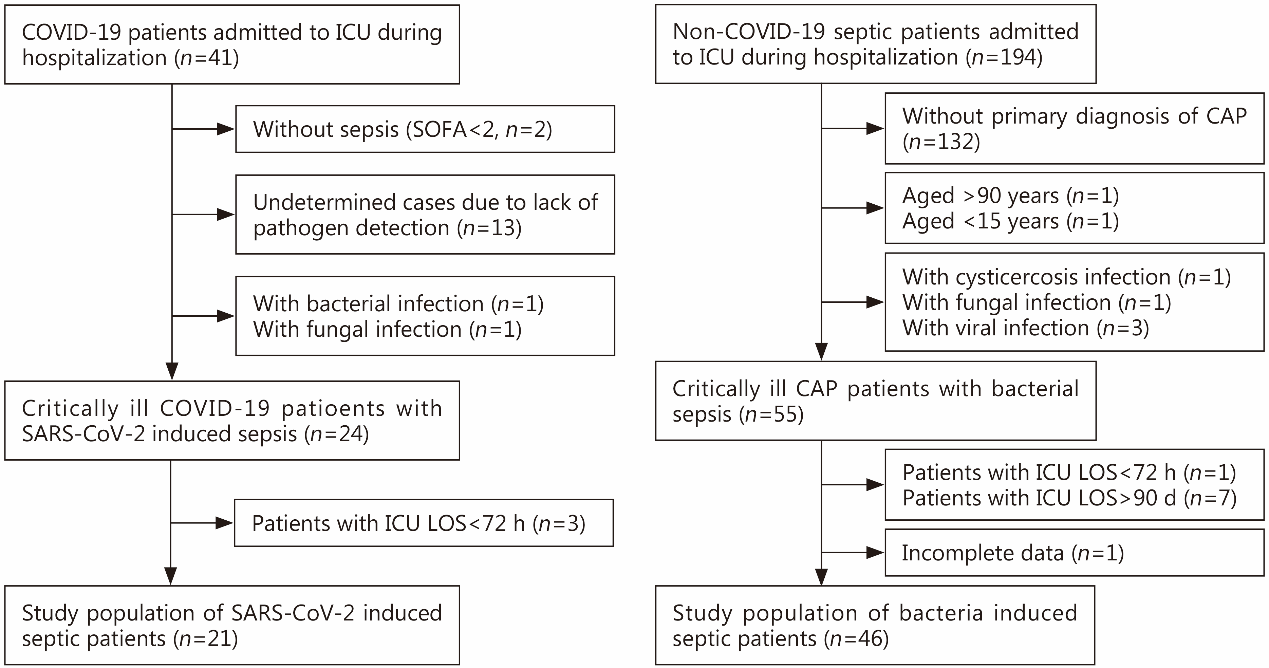
**

**Appendix Figure 1. Flow diagram of patient inclusion.**

COVID-19: Coronavirus disease 2019; SARS-CoV-2: Severe acute respiratory syndrome coronavirus 2; SOFA: Sequential organ failure assessment; ICU: Intensive care unit; LOS: Length of stay; CAP: Community-acquired pneumonia.
